# Case Report: Generalised Panniculitis as a Post-COVID-19 Presentation in Aicardi-Goutières Syndrome Treated With Ruxolitinib

**DOI:** 10.3389/fped.2022.837568

**Published:** 2022-04-25

**Authors:** Abirami Pararajasingam, Rachel E. Bradley, Jennifer Evans, Ashima Lowe, Richard Goodwin, Stephen Jolles

**Affiliations:** ^1^Department of Dermatology, Aneurin Bevan University Health Board, Newport, United Kingdom; ^2^Immunodeficiency Centre for Wales, University Hospital of Wales, Cardiff, United Kingdom; ^3^Department of Paediatrics, University Hospital of Wales, Cardiff, United Kingdom

**Keywords:** Aicardi-Goutières syndrome, COVID-19, ruxolitinib, panniculitis, SAMHD1 mutation

## Abstract

Aicardi-Goutières syndrome (AGS) is a rare hereditary early-onset encephalopathy. The syndrome was first described in 1984, and is characterised by upregulation of the type I interferon (IFN) pathway, which is involved in the host immune response against viral infections, including SARS-CoV-2. Whilst defects in type I IFN pathways have been described in association with severe coronavirus disease 2019 (COVID-19), less is known about the outcomes of upregulation. We describe an unusual case of generalised panniculitis as a post-COVID-19 phenomenon in a child with AGS. Our patient was initially managed with systemic steroid therapy, but due to relapse of symptoms on weaning, an alternative therapy was sought. In this case, a novel use of ruxolitinib, a JAK inhibitor, has resulted in lasting remission without complications. We discuss the probable protective role of IFN upregulation following COVID-19 infection in AGS and possible immunological mechanisms driving the panniculitis and therapeutic response in our case.

## Introduction

Aicardi-Goutières syndrome (AGS) was first described by Aicardi and Goutières in their report of eight children from five families with an early-onset encephalopathy characterised by basal ganglia calcification, white matter abnormalities, and a chronic cerebrospinal lymphocytosis (1). There is a typical elevation of interferon (IFN) alpha levels in both cerebrospinal fluid (CSF) and serum of these patients (2). More recently, evidence of abnormal IFN activity was demonstrated by identifying an “interferon signature” in the peripheral blood. This measures the expression of IFN-stimulated genes (e.g., IFI27, IFI44L, IFIT1, ISG15, RSAD2, and SIGLEC1) and has been identified in patients with mutations in genes associated with AGS (3, 4).

Type I IFN was originally described as a soluble factor, produced by cells treated with inactivated non-replicating viruses, that subsequently successfully blocked or interfered on infection with the live virus (5, 6). The rapid induction and amplification of the type I IFN system is a key component of antiviral immunity (e.g., against the novel coronavirus) which activates the JAK-STAT signalling pathway and transcription of IFN-stimulated genes (7).

In AGS, the main extraneurological symptoms are chilblain-like cutaneous lesions in up to 40% of cases, usually on the fingers, toes, and ears (8). Some children with AGS also develop symptoms overlapping with systemic lupus erythematosus (SLE) (9). There appears to be a phenotypic overlap between AGS, *in utero* HIV-1 infection, and SLE, suggesting that these result from the common pathological feature of type I IFN upregulation (10). Mutations in nine genes, namely, TREX1 (AGS1), RNASEH2A (AGS2), RNASEH2B (AGS3), RNASEH2C (AGS4), SAMHD1 (AGS5), ADAR1 (AGS6), IFIH1 (AGS7), LSM11, and RNU7-1, have been identified as the causative agent of AGS (4, 11). These genes are involved in the DNA damage response and sensing free cytosolic DNA/RNA. Defects in these genes result in an inappropriate innate immune response, triggering an increased secretion of the antiviral cytokine type I IFN and upregulation of IFN-stimulated genes, ultimately responsible for the main features of the disease (12). At a cellular level, this resembles the type I IFN response following the exposure to viral DNA or RNA, thus explaining why the AGS phenotype may mimic that of viral infections. However, in contrast to viral infection, AGS is considered to represent an abnormal response to endogenous or self-derived nucleic acids (10).

Genotype-phenotype correlation has been observed, and there are certain core features that overlap across mutations in the nine genes with the vast majority of affected subjects displaying severe motor and cognitive impairment (13). In a large cohort of 252 patients with AGS, the most common clinical features were identified as cognitive impairment, dystonia, microcephaly, and chilblain lesions reported in 92, 75, 63, and 42% of cases, respectively (14). Chilblain lesions are the most well-documented extraneurological sign. These cutaneous manifestations are characterised by areas of inflammation and intermittent necrosis, found mainly on the fingers, toes, pressure points, and ears (13). Acrocyanosis is also very frequent, as is periungual erythema (15).

The course of AGS is variable; most affected individuals present around 4 months of age with a subacute onset encephalopathy followed by marked developmental delay/regression as well as signs of neurological impairment and a slowing of head growth over a few months (8). Early mortality is described in some, not long following symptom onset, whilst others go on to have a more stable clinical course, without significant further deterioration (16).

AGS is a rare genetic disease, and as such, treatment approaches are based on case series with limited clinical trial data. There is no cure for AGS, and treatments aim to limit the symptoms. As the pathogenesis of AGS is related to inappropriate activation of the innate immune response, generalised immunosuppression with high-dose corticosteroid therapy has been trialled and targetted therapies such as reverse transcriptase inhibitors and JAK inhibitors have been used.

Whereas genetic and acquired defects in the type I IFN pathway have been shown to predispose to more severe outcomes in coronavirus disease 2019 (COVID-19) (17–20), the consequences of this infection in the setting of upregulated type I IFN signalling are less well understood. We report a case of a child with AGS presenting acutely with generalised panniculitis as a post-COVID-19 phenomenon with a beneficial response to the ruxolitinib, a JAK 1/2 inhibitor.

## Case Description

Our patient was born to consanguineous parents from Pakistan and had a confirmed diagnosis of AGS with previous genetic testing confirming homozygous SAMHD1 mutations (c. 427C > T). She had an older sibling who was confirmed to have the same genotype and who displayed an almost identical phenotype, manifesting in severe neurological deficit, spastic quadriplegia, and dystonia. Her regular medication consisted of sodium valproate (160 mg twice daily) and baclofen. Although the older sibling had developed cutaneous manifestations in the form of acral perniosis and periungual necrosis at the age of 8 years, there were no prior cutaneous changes in our patient.

At 5 years of age, the patient was admitted with a 3-week history of cutaneous changes affecting her face and upper limbs but was otherwise well. Her grandfather, who co-habited with the family, had developed a febrile illness and tested positive for COVID-19 by PCR a few weeks previously. Despite this, she had no history of fever, cough, or other systemic symptoms. No other family member was unwell, including the patient’s sibling, who remained well and completely asymptomatic.

**FIGURE 1:**
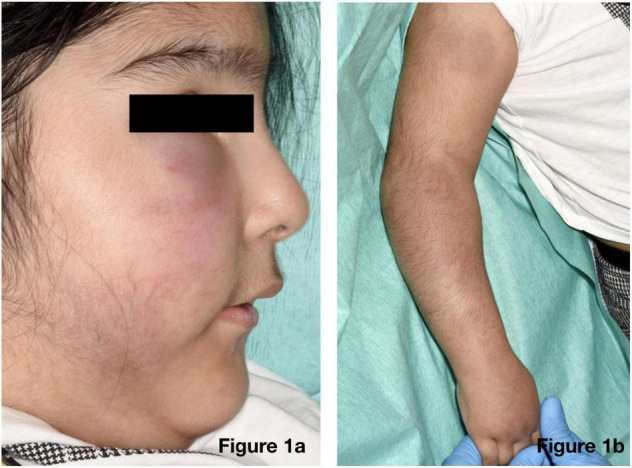
Physical examination revealed dusky indurated plaques on the central and lower face **(a)** and arms **(b)** with associated oedema.

On physical examination of the new skin changes, there were dusky areas of induration involving the central and lower face, bilateral forearms, and extending onto her hands with associated oedema (Figure 1). There was notable sparing of dependent areas, including the back and posterior legs. Scattered petechial macules were also noted on the left forearm and foot. Aside from the cutaneous changes, no other extraneurological signs were found on examination. She was afebrile and the remaining observations were within the normal range. Investigations demonstrated a mild transaminitis with an ALT of 49 U/L (9–25), a raised amylase of 153 U/L (25–101), D-dimer of 1,785 μg/L (<500), and lactate dehydrogenase of 525 U/L (192–321). Throat swabs for SARS-CoV-2 RNA PCR were negative. Of note, chest radiography, renal function, autoimmune studies, and C-reactive protein levels were normal; however, there was a significant elevation in the terminal complement complex (TCC) at this stage at 244 ng/ml (0–80).

**FIGURE 2:**
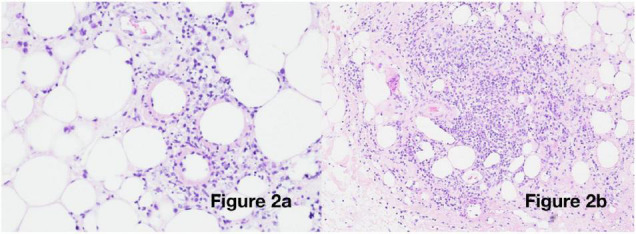
Histological examination: Incisional biopsy from the right upper arm [haematoxylin and eosin (H&E) staining] showing **(a)** 20× magnification: vasculitis and fibrinoid necrosis and **(b)** 10× magnification: deep subcutaneous infiltrate with oedema and karyorrhexis.

Suspicion of a post-COVID-19 phenomenon was supported, with SARS-CoV-2 antibody testing demonstrating immunoglobulin G (IgG) positivity (in the absence of vaccination). Her cutaneous changes continued to progress, spreading across the chest, arms, and thighs, without any corresponding deterioration in general health. An incisional biopsy revealed a predominantly lymphocytic, nodular, perivascular, and periadnexal inflammatory infiltrate in the deep dermis and subcutis, with associated oedema, fat necrosis, and karyorrhexis in the subcutaneous fat, confirming a panniculitis (Figure 2). Viral particles were not identified on electron microscopy. Neither the patient nor her sister had previous evidence of panniculitis.

She was commenced on prednisolone 20 mg (1 mg/kg) once daily, and within a few days, there was an improvement in the dusky discolouration across the face, limbs, and chest, with near-resolution and softening of the panniculitis observed after a few weeks. As the prednisolone dose was weaned to 0.25 mg/kg/day, the panniculitis relapsed and the higher dose was promptly reintroduced, with good effect. Further attempts at dose reduction were met with a similar deterioration, and although she remained systemically well, the lesions were associated with significant discomfort. Lymphocyte subsets taken 4 months following the onset of rash and 3 months following the trials of steroids showed CD3+ (T cells) 2,780 × 10^6^ cells/L (900–4,500) and CD19+ (B cells) 3,690 × 10^6^ cells/L (200–2,100).

**FIGURE 3:**
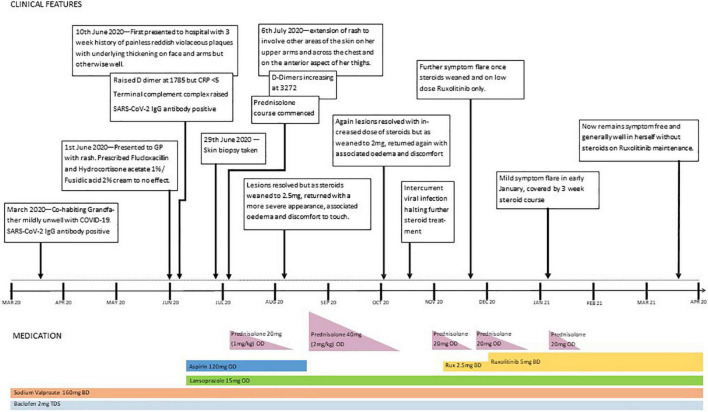
Timeline with the most relevant data of the clinical case.

Ruxolitinib, a JAK1/2 inhibitor, was commenced, building up to 5 mg twice daily. The rash improved and oedema resolved, and it was possible to wean off systemic steroids. Unfortunately, 10 days after stopping prednisolone, some oedema and discomfort reappeared, without any skin discolouration. Although this was a much less severe relapse than before, it necessitated the short-term re-introduction of prednisolone, alongside the ruxolitinib. A second attempt at steroid withdrawal was successful, and 4 months on, our patient remains stable on ruxolitinib alone. A timeline of events is shown in Figure 3.

## Discussion

Our patient had an asymptomatic COVID-19 illness with the development of detectable antibody response and the post-infection cutaneous manifestations of panniculitis in the setting of AGS. A recent report documenting the course of COVID-19 in 94 patients with inborn errors of immunity described three patients with AGS including our patient (21). While numbers are very limited, it is of interest that all three, including the two other patients with biallelic RNASEH2B variants, had completely asymptomatic “silent” COVID-19 infections possibly suggesting a protective effect of IFN upregulation in the setting of viral infection. Indeed, one recent case report shows increased IFN levels from baseline in a patient with AGS suffering from mild COVID-19 illness (22).

This theory concurs with studies demonstrating that disruption of type I IFN signalling is associated with severe COVID-19 illness. In a study, 23 of 659 (3.5%) patients with life-threatening COVID-19 pneumonia displayed genetic defects abolishing induction or amplification of type I IFN (23). A study of 50 adult patients with COVID-19 showed that IFN levels were significantly reduced in critically unwell patients compared to those with mild to moderate infection with the suggestion that this resulted in uncontrolled proliferation of the virus (24). Similarly, the examination of post-mortem COVID-19-infected lung tissue demonstrates that SARS-CoV-2 evokes a distinctive transcriptional response, which lacks type I and type III IFN expression (17–20). In addition, it has been noted that of 987 individuals with life-threatening COVID-19 infection, 13.7% had autoantibodies against type I IFNs detected (25).

It is well reported that children with COVID-19 generally present with milder disease. This may be due to a prompt innate immune response, where IFN signalling plays a key role, which is more frequently dealing with respiratory viruses and vaccinations that children are regularly exposed to in early life (26). However, few children go on to develop the post-infectious multi-system inflammatory syndrome (MIS-C), usually manifesting a number of weeks following the initial infection. One study found that 3/18 patients with MIS-C had genetic mutations affecting the regulation of IFN signalling (27).

Although our patient did not report any typical symptoms of COVID-19 infection, she subsequently manifested persistent skin lesions over her face, limbs, and chest at a time when the SARS-CoV-2 PCR was negative with positive SARS-CoV-2 serology. The sparing of dependent areas observed in our case is suggestive of a chilblain-like process, which is a recognised cutaneous feature of COVID-19 (19) and a hallmark feature of genetic type I interferonopathies like AGS (17–20, 28). This is likely an inflammatory process mediated by IFN in an individual that is genetically prone to such immune dysregulation that has been triggered by the initial viral infection.

From a physiological perspective, chilblains occur often due to cold-induced constriction of small arteries and veins, hence usually manifesting on distal extremities. In our patient, her immobility and supine positioning may function to protect the posterior aspect of her torso and limbs from temperature drops and partly explain the sparing of these sites. In the context of COVID-19, chilblain-like lesions (perniosis) characteristically occur a few weeks following the acute infection which is often mild or asymptomatic (29). Typical histopathological features, which were seen in our patient, include a perivascular lymphocytic infiltrate with dermal oedema. However, in addition to these classical histological features of perniosis, our patient also had more extensive involvement of the subcutaneous fat, in keeping with a panniculitis. Panniculitis with or without lipodystrophy has been reported in children with type I interferonopathies (30).

So far, generalised panniculitis is an unreported cutaneous feature of COVID-19, and it may therefore be possible that the combination of infection with COVID-19 on a background of underlying AGS with associated IFN upregulation resulted in the observed prolonged panniculitis. Indeed, a number of reports have suggested that the mechanism behind chilblain-like lesions in the setting of SARS-CoV-2 infection is IFN-driven (31–33). In addition, there are a number of case reports describing panniculitis at the administration site of IFN therapy and associations with systemic therapy (34–41), suggesting a direct role for IFN in the development of panniculitis. The delayed manifestation of the IFN-driven inflammatory process observed, whilst limited to the skin, may be reflective of delayed MIS-C seen in children with loss-of-function mutations associated with enhanced IFN signalling (42).

Treatment of the cutaneous manifestations in our patient was initially responsive to high-dose corticosteroid therapy. A number of reports of treatment of AGS with steroids confirm a reduction in IFN-alpha levels in the CSF but no clinical improvement (43–46). More recently described is a case where neuroradiological changes on MRI showed an improvement after high-dose steroids and IV immunoglobulin (47). When steroid weaning failed to maintain remission, therapy targetted against IFN and its signalling pathways directly was initiated in the form of a JAK inhibitor which proved effective for the patients cutaneous symptoms. There are encouraging results from a number of publications using the JAK inhibitors, ruxolitinib (48–52) and baricitinib (53), in type I interferonopathies and in AGS specifically with some neurological and developmental improvement reported with ruxolitinib. In addition, reports of the first trial of baricitinib in 35 patients with AGS showed similar improvements. A phase 2 trial of baricitinib in AGS is now currently underway (ClinicalTrials.gov Identifier: NCT03921554).

## Conclusion

We suggest that our patient developed COVID-19-triggered, prolonged auto-inflammatory, type I IFN-driven panniculitis, resulting from the exceedingly rare combination of AGS and COVID-19 infection. Therapy was challenging as the panniculitis was responsive only to very high doses of steroids and relapsed on tapering the dose. The novel use of ruxolitinib, used to both control the panniculitis and spare steroids, was effective and well tolerated, and it will be important to determine if ongoing therapy is needed.

## Data Availability Statement

The original contributions presented in the study are included in the article/supplementary material, further inquiries can be directed to the corresponding author/s.

## Ethics Statement

Written informed consent was obtained from the individual(s), and minor(s)’ legal guardian/next of kin, for the publication of any potentially identifiable images or data included in this article.

## Author Contributions

All authors listed have made a substantial, direct, and intellectual contribution to the work, and approved it for publication.

## Conflict of Interest

The authors declare that the research was conducted in the absence of any commercial or financial relationships that could be construed as a potential conflict of interest.

## Publisher’s Note

All claims expressed in this article are solely those of the authors and do not necessarily represent those of their affiliated organizations, or those of the publisher, the editors and the reviewers. Any product that may be evaluated in this article, or claim that may be made by its manufacturer, is not guaranteed or endorsed by the publisher.
